# A nomogram to predict the probability of passing the American Board of Internal Medicine examination

**DOI:** 10.3402/meo.v17i0.18810

**Published:** 2012-10-16

**Authors:** Andrei Brateanu, Changhong Yu, Michael W. Kattan, Jeff Olender, Craig Nielsen

**Affiliations:** 1 Department of Medicine, Cleveland Clinic, Cleveland, Ohio, USA; 2 Department of Quantitative Health Sciences, Cleveland Clinic, Cleveland, Ohio, USA

**Keywords:** board examination, in-training examination, internal medicine, residents, program directors

## Abstract

**Background:**

Although the American Board of Internal Medicine (ABIM) certification is valued as a reflection of physicians’ experience, education, and expertise, limited methods exist to predict performance in the examination.

**Purpose:**

The objective of this study was to develop and validate a predictive tool based on variables common to all residency programs, regarding the probability of an internal medicine graduate passing the ABIM certification examination.

**Methods:**

The development cohort was obtained from the files of the Cleveland Clinic internal medicine residents who began training between 2004 and 2008. A multivariable logistic regression model was built to predict the ABIM passing rate. The model was represented as a nomogram, which was internally validated with bootstrap resamples. The external validation was done retrospectively on a cohort of residents who graduated from two other independent internal medicine residency programs between 2007 and 2011.

**Results:**

Of the 194 Cleveland Clinic graduates used for the nomogram development, 175 (90.2%) successfully passed the ABIM certification examination. The final nomogram included four predictors: In-Training Examination (ITE) scores in postgraduate year (PGY) 1, 2, and 3, and the number of months of overnight calls in the last 6 months of residency. The nomogram achieved a concordance index (CI) of 0.98 after correcting for over-fitting bias and allowed for the determination of an estimated probability of passing the ABIM exam. Of the 126 graduates from two other residency programs used for external validation, 116 (92.1%) passed the ABIM examination. The nomogram CI in the external validation cohort was 0.94, suggesting outstanding discrimination.

**Conclusions:**

A simple user-friendly predictive tool, based on readily available data, was developed to predict the probability of passing the ABIM exam for internal medicine residents. This may guide program directors’ decision-making related to program curriculum and advice given to individual residents regarding board preparation.

## Introduction

The American Board of Internal Medicine (ABIM) evaluates and certifies the knowledge, judgment, and skills of the physicians and their ability to deliver high-quality care (1). To become certified, physicians need to meet the graduate medical education training requirements, demonstrate clinical competence in the care of patients, and pass the certification examination in internal medicine, a secure exam that is administered by ABIM at the end of the internal medicine residency training (2). The Accreditation Council for Graduate Medical Education (ACGME), who is responsible for the accreditation of medical training programs within the United States requires that ‘a program's graduates must achieve a pass rate on the certifying examination of the ABIM of at least 80% for first time takers of the examination in the most recently defined three-year period’ (3).

There is no mathematical model in the literature for predicting the probability of passing or failing the ABIM examination by internal medicine graduates. The first objective of our study was to develop a nomogram based on resident characteristics from one internal medicine residency program and assess its accuracy in predicting the graduates’ successful passing of the ABIM certification examination. The second objective was to test the validity of the nomogram using data from two other independent residency programs.

## Methods

### Design, subjects

The development cohort consisted of 194 residents who successfully completed their training in internal medicine at the Cleveland Clinic, Ohio, between 2004 and 2008. Data were collected from the residency program records and de-identified. The validation cohort was compiled retrospectively in 2012, using 126 residents who graduated between 2007 and 2011 from two other residency programs in Cleveland, Ohio. The study was reviewed and approved by the local institutional review board.

### Baseline characteristics

Data were collected on selected background independent variables, including resident age, gender, type of medical school (conferring medical doctor (MD) or doctor of osteopathy (DO) degrees), time passed between medical school graduation and the start of the residency program, fellowship aspiration when entering the residency, United States Medical Licensing Examination (USMLE) step 1, 2, Clinical Knowledge (CK), and step 3 scores, and the assessment score received by participants at the time they were interviewed for obtaining a position in the residency program. Each candidate to the Cleveland Clinic residency program was interviewed by two faculty members and scored on a scale of 1 (worst) to 10 (best). The final score was calculated by averaging the scores obtained from each interviewer. If the difference between the two scores exceeded two points, the candidate was reviewed by a third faculty and a final score assigned. In addition, information on the dependent variables, namely, noon conference attendance, In-Training Examination (ITE) scores (percentage (%) correct) in the postgraduate year (PGY) 1, 2, 3, and the number of months with overnight calls during the last 6 months of the residency, were also collected.

### Primary outcome variable

The primary outcome in both development and validation cohorts was passing the ABIM examination in the first attempt.

### Statistical analysis

In the development cohort, multivariable logistic regression analysis was conducted to investigate the predictive power of each individual characteristic on the ABIM exam performance. Missing values in the variables were imputed before the multivariable analysis by the algorithm of multivariate imputation by chained equations (MICE) (4), with the purpose of eliminating the selection bias if only residents with complete data were used. All variables were included for initial evaluation except the USMLE 3 scores, where more than half of the values were missing. Forward stepwise selection (FSS) with the Akaike information criterion (AIC) was used to select a subset of the predictors that maximize the AIC of the final model, on which a nomogram was built for predicting the likelihood of passing the ABIM exam. The FSS with the AIC process along with the final model was internally validated with 1,000 bootstrap resamples, where the predictive performance of the nomogram on the future new residents was assessed after correcting the over-fitting bias. The predictive accuracy was quantified by concordance index (CI) (5), which is identical to the area under the receiver operating characteristic (ROC) curve with a range of 0.5 (no predictive power) to 1 (perfect prediction). Moreover, the performance of the nomogram in the validation cohort was assessed through CI and calibration. The calibration that describes how far the nomogram predictions are from the actual outcomes, that is, the 45 degree line was visually checked. First, resident graduates were divided into quintiles based on their predicted passing probabilities. Then the mean predicted passing probabilities were plotted against the actual passing rate within each quintile to check the correspondence.

A critical *p*-value of 0.05 was used to determine the statistical significance for all hypothesis tests. All statistical analyses were performed using the open source software R version 2.12.2 (6) with additional packages: mice, Design, and Hmisc.

## Results

Baseline characteristics are described in detail in Table 1. Of the 194 graduates in the development cohort, 175 (90.2%) passed the ABIM examination. At the time of joining the internal medicine residency, the mean age of the Cleveland Clinic residents was 28.6 years and two-thirds were males. Most residents were MD graduates (80.4%), with a smaller percentage coming from osteopathic schools (19.6%). The mean time passed between the medical school graduation and beginning of the residency program was 1.7 years. There was a high interest in pursuing a fellowship career in more than 80% of the residents. The residents who passed the ABIM exam appeared to have higher interview scores than the ones that failed the exam. There was no substantial difference between the two groups with regard to the noon conference attendance or number of months with calls in the last 6 months of the residency. The scores in the USMLE 1 and 2 CK, as well as in the ITE 1, 2, and 3 seemed higher in the group that passed the ABIM examination.

**Table 1:** Baseline graduates characteristics

Variables	Total cohort, *N* = 194	Graduates with missing data, *n* (%)	ABIM failure, *N* = 19 (9.8)	ABIM pass, *N*=175 (90.2)	*P*
Age in years	28.6±3.6	0	29.1±4.3	28.6±3.5	0.539
Male sex	129 (66.5)	0	9 (47.4)	120 (68.6)	0.063
MD graduates	156 (80.4)	0	14 (73.7)	142 (81.1)	0.437
Fellowship aspiration	162 (83.5)	0	16 (84.2)	146 (83.4)	1.000
Break in years*	1.7±2.5	0	0.4±0.9	1.8±2.6	0.023
USMLE 1 score	228.1±17.6	21 (10.8)	209.0±13.6	230.0±16.8	<0.001
USMLE 2CK score	232.2±19.8	25 (12.9)	211.2±15.7	234.2±19.0	<0.001
USMLE 3 score	212.9±13.5	145 (74.7)	216.0±NA	212.8±13.6	0.817
Interview score	8.0±1	14 (7.2)	7.2±0.9	8.1±1	0.001
Noon conference attendance†	60±10	1 (0.5)	50±10	60±10	0.069
ITE‡ score in PGY1	59.9±8.0	10 (5.1)	50.5±5.9	61.0±7.5	<0.001
ITE‡ score in PGY2	62.5±9.4	12 (6.2)	50.2±5.8	63.9±8.7	<0.001
ITE‡ score in PGY3	65.9±7.5	9 (4.6)	54.1±5.4	67.3±6.4	<0.001
Number of call months in the past 6 months	2.0±0.9	0	2.3±0.9	2.0±0.9	0.089

Values expressed as mean±SD or number (percent).

* Time passed between the medical school graduation and beginning of the residency program.

† Attendance expressed as percentage.

‡ In-Training Examination score expressed as percentage of questions answered correctly.

Abbreviations: ABIM=American Board of Internal Medicine; MD=medical doctor; USMLE=United States Medical Licensing Examination; CK=Clinical knowledge; ITE=In-Training Examination; PGY=postgraduate year.

The multivariable logistic regression model was developed to predict a binary outcome of the ABIM status (pass/fail). Of the 14 predictors considered for the analysis, only 13 were included. The scarcity of data on USMLE 3 scores precluded the addition of this variable to the initial model (missing information on 145 residents). This is due to the residents not required to report the USMLE 3 score to the residency program. Four factors ITE 1, 2, 3, and number of call months in the last 6 months of the residency were found to contribute to the prediction model (Table 2).

**Table 2:** The final multivariable logistic regression analysis, on which the nomogram was based

Predictor variables	Odds ratio (95% CI)	*P*
ITE* score in PGY1	1.27 (1.02, 1.58)	0.031
ITE* score in PGY2	1.19 (1.07, 1.33)	0.002
ITE* score in PGY3	1.58 (1.23, 2.04)	<0.001
Number of call months in the past 6 months	0.34 (0.11, 1.06)	0.065

* In-Training Examination score expressed as percentage of questions answered correctly.

**Fig. 1:**
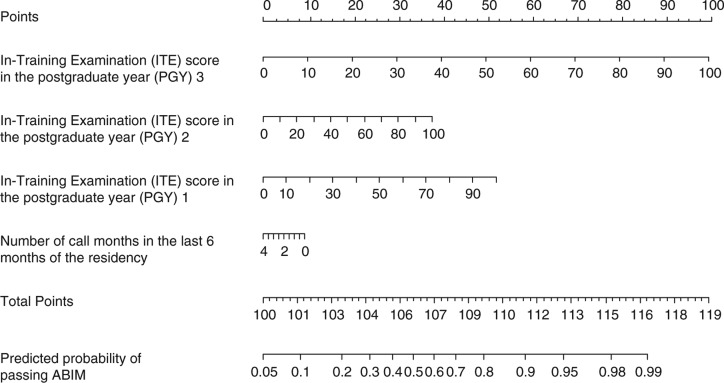
Predictive graphic nomogram for the probability of passing the American Board of Internal Medicine (ABIM) exam.

**Fig. 2:**
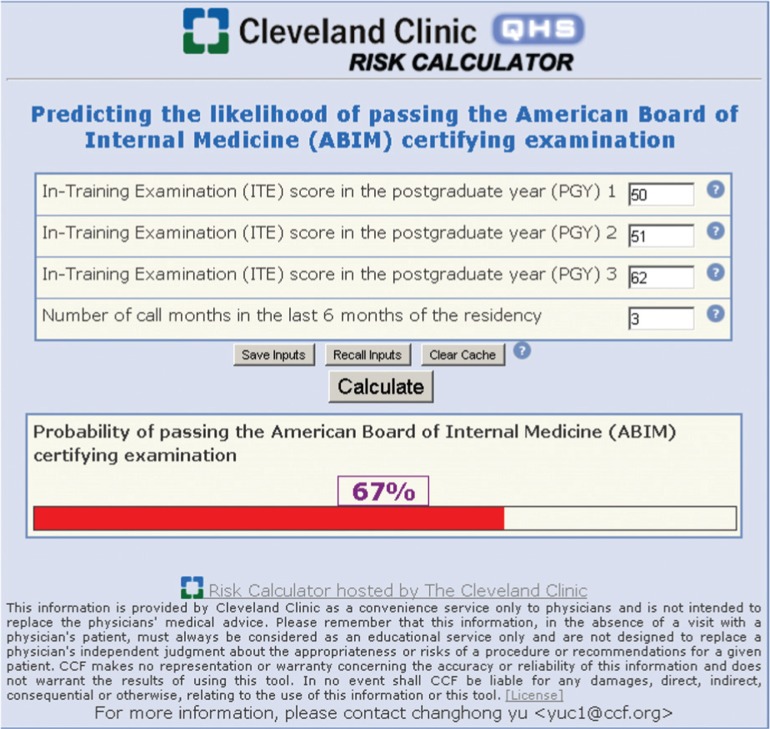
Screenshot of nomogram Web site.

The graphic nomogram derived from the final regression model is presented in Fig. 1. To predict the probability of passing the ABIM examination, the nomogram is used by initially locating the scores on each of the ‘ITE 1, 2, 3’ and ‘number of call months’ horizontal scales and drawing vertical lines up to the uppermost ‘points’ scale. When the points generated from each of the four factors are added together, the sum is then plotted on the lower ‘total points’ scale, and the predicted probability is found on the corresponding lowermost scale by drawing a vertical line down to the ‘predicted probability of passing ABIM’ scale. An online predictive tool (http://rcc.simpal.com/RCEval.cgi?RCID=LbGdeA) (Fig. 2) was created to facilitate the automatic calculation of the predicted probability for easier use.

The CI based on the internal validation on the development cohort was 0.98 (95% CI, 0.95–1.00).

### Nomogram external validation

**Fig. 3:**
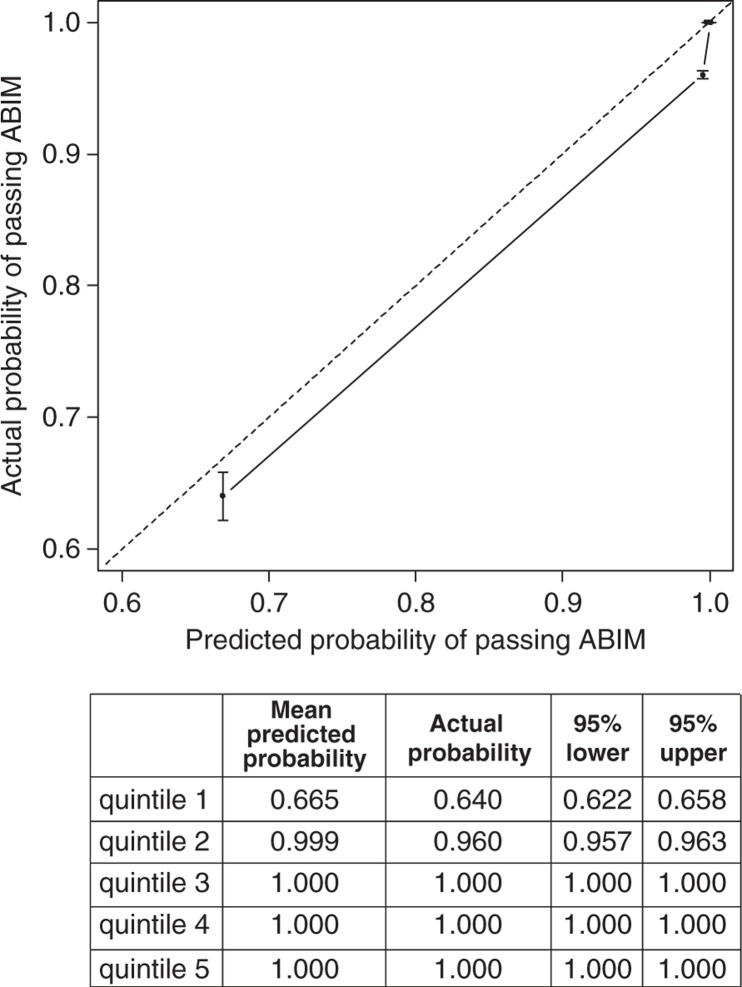
Calibration plot for predicted and observed passing ABIM examination. Residents in the validation cohort were divided in five quintiles based on the predicted probability. The 45-degree straight line represents ideal agreement between actual and predicted probability. The vertical bars represent the 95% CI of the actual probability. Note that the points for the three highest quintiles were overlapping together in the plot because of ignorable differences.

Of the 126 graduates from the other two residency programs used for external validation, 116 (92.1%) passed the ABIM examination. The calibration plot was generated by grouping the validation cohort into five subgroups, sorted by the predicting passing probabilities and plotting the mean predicted probabilities against the actual fraction of residents within each quintile (Fig. 3). The nomogram CI in the validation cohort was 0.94 (95% CI, 0.90–0.99). When compared with each of the four individual predictors included in the validation set, the nomogram performed better than the ITE 1, 2, 3 scores and the number of call months in the last 6 months of the residency. The ITE 3 score was the most important predictor, which was reflected in the nomogram as well (Table 3). The correspondence between the actual passing rate and the nomogram predictions suggests a good calibration of the nomogram in the validation cohort. The model has a very good prediction for graduates with high likelihood to pass the exam and slightly overestimates the passing rate for those with low likelihood.

**Table 3:** Concordance index (CI) and 95% confidence intervals for each of the four individual predictors along with the multivariable regression model

Predictors	Concordance index	Lower 0.95	Upper 0.95
Nomogram	0.943	0.889	0.983
ITE* score in PGY1	0.839	0.751	0.913
ITE* score in PGY2	0.872	0.793	0.940
ITE* score in PGY3	0.940	0.855	0.988
Number of call months in the past 6 months	0.570	0.387	0.726

* In-Training Examination score expressed as percentage of questions answered correctly.

## Discussion

We have created a nomogram that may be used to help residency programs to predict the probability of an individual resident passing the ABIM examination on their first attempt. To our knowledge, this is the first tool to accurately predict the chance of success on the ABIM examination. It has the advantage of using common predictors that are available to all the internal medicine residents and program directors.

United States Medical Licensing Examination is a three-step examination with four tests, sponsored by the Federation of State Medical Boards and the National Board of Medical Examiners (7). Passing the first two steps is required before graduates start their postgraduate training, while passing the third step is required before graduation from the residency, to obtain a license to practice medicine in the United States (8). ITE was developed in 1988 by the American College of Physicians, the Association of Professors of Medicine, and the Association of Program Directors in Internal Medicine to help program directors assess the residents and identify potential knowledge deficiencies (9).

The initial univariate analysis (Table 1) in the development cohort showed a strong correlation between the results of the ABIM examination and the USMLE 1 and 2 CK scores, the assessment score received by participants at the time they were interviewed for obtaining a position in the residency program, and the ITE scores in the PGY 1, 2, and 3. There was also a weak association with the time passed between the medical school graduation and beginning of the residency program, suggesting maybe that candidates having more time to study or getting some experience before starting the residency have a higher chance of succeeding in the ABIM exam. The USMLE scores and the interview scores did not portend the ABIM results in the final multivariable logistic regression model, as ITE and numbers of calls in the last 6 months of residency training had a more predictive weight. This could actually be explained by the fact that residents included in the study have already been selected based on the USMLE scores. Regarding the interview score, some of the determinants of this score take into account precisely the USMLE scores.

Previously, few studies looked at the association between the board results and ITE scores during the second (10–13) or third (10) year of internal medicine training. Similarly, our study found a very good CI for the ITE scores in the PGY 2 and 3 years (Table 3) and is the first one to show that actually the ITE results during every training year (PGY 1, 2 and 3) are important in predicting the board results and provide an estimated probability in passing the ABIM exam. The ITE score during PGY 3 had the highest weight in predicting the probability score. This has very important implications for the residents considering that the ITE takes place every year during fall. Knowing the score in advance could make them change their study habits and improve the chance of passing the ABIM exam. In addition, it would give program directors the ability to identify the residents who are likely to benefit the most from additional board review curriculum.

Other studies looked at the correlation between the ability to pass the board examination and other criteria, including background resident features, namely age and gender, time passed between medical school graduation and starting the residency, USMLE scores, interest in pursuing fellowships, and attendance to teaching conferences (14–16). Although in the above mentioned studies, USMLE scores, analyzed separately, were associated with the passing of the board examination, there is no mathematical model in the literature on how to predict the ability to pass the ABIM exam when the USMLE, ITE scores, and other resident individual characteristics are analyzed together.

The ACGME requires residents to attend the regularly scheduled didactic sessions (3). Our study confirmed the FitzGerald findings (14) that there is no association between residents’ attendance to conferences and the board score results. This cannot be generalized to other programs but has implications for the type and content of medical topics presented during these conferences. It also suggests that conferences cannot replace the individual effort each resident invests into studying.

We found no difference between residents from osteopathic and allopathic schools in the success rate of passing the board examination. The background resident characteristics, namely age and sex, did not influence the success either, but the resident age distribution was narrow, therefore findings cannot be generalized to other programs with a different age distribution.

Residents fellowship aspiration did not correlate with the board pass rate, suggesting that although residents interested in subspecialties might have extra elective rotations in their favorite specialty, they receive an equally effective training when compared to the ones planning to pursue a carrier in general internal medicine.

An interesting finding is the influence that the number of calls during the past 6 months of residency has upon the ABIM probability of success. Although small in comparison to the ITE score relevance, it suggests that easier rotation months at the end of the residency give residents an additional advantage and increases their chance of passing the board. This could be effectively used by the program directors, when scheduling rotations for the residents.

The strength of the article resides in the fact that the nomogram uses simple variables common to all the residency programs and is validated with an external cohort of residents from two other independent internal medicine residency programs.

A limitation of our study is that we had the ABIM examination as a binary outcome variable (pass/fail) as it is known that there is a wide range of variance both below and above the cut-off passing score. Having the actual passing score and knowing which residents will be immediately above the cut-off point might help target specific interventions during residency training that will help promote the resident from a competent to an outstanding one. Similarly, the ability to show which resident will be immediately below the threshold might help motivate them and intensify the efforts toward personal improvement. However, from a career standpoint, passing the ABIM examination and not the score is the most important outcome.

This article has also several limitations common to retrospective studies. We did not have enough data on USMLE step 3 results to conclude on the importance of this variable. We used noon conference, but not morning report or grand rounds attendance, because of insufficient recorded data. In addition, the nomogram itself has some limitations, having good prediction for the residents with high likelihood of passing the ABIM examination but slightly overestimating the passing rate for those with low likelihood. Due to these limitations, we feel that a larger sample of residents, the possibility of additional novel predictors that we did not include, and data collection from different residency programs are likely to improve the nomogram's accuracy and portability.

In conclusion, we created and validated a simple tool that predicts the probability of internal medicine residents passing the ABIM examination. The nomogram has the potential to help programs design curricular activities to support at-risk residents preparing for the ABIM examination.
